# Revisiting *CDKN2A* dysregulation in Ewing sarcoma

**DOI:** 10.1002/1878-0261.70008

**Published:** 2025-03-13

**Authors:** Anjali Paragji, Vivek Shastri, Elham Nasri, John A. Ligon, Leighton A. Elliott, Paul Castillo‐Caro, Jatinder K. Lamba, Elias J. Sayour, Nathan D. Seligson

**Affiliations:** ^1^ Department of Pharmacotherapy and Translational Research, College of Pharmacy The University of Florida Jacksonville FL USA; ^2^ Department of Pathology, College of Medicine The University of Florida Gainesville FL USA; ^3^ Department of Pediatrics, College of Medicine The University of Florida Gainesville FL USA; ^4^ Department of Neurosurgery, College of Medicine The University of Florida Gainesville FL USA; ^5^ Department of Pharmacogenomics and Translational Research Nemours Children's Health Jacksonville FL USA

**Keywords:** biomarker, next‐generation sequencing, prognosis

## Abstract

Ewing sarcoma (EwS) is a rare and aggressive malignancy, which frequently affects children. One of the few recurrent genomic variants in EwS is genomic copy number deletion of *CDKN2A*; however, the clinical consequences of dysregulation of *CDKN2A* in EwS are unclear. In this study, we revisit *CDKN2A* to investigate its role as a potential prognostic biomarker in EwS using data from EwS pre‐clinical models as well as clinical samples from patients with EwS. We demonstrate the potential essentiality of *CDKN2A* dysregulation and sustained downstream *CDK4*/*CCND1* activity. Finally, we present evidence that high expression of *CDKN2A* is a negative prognostic biomarker at diagnosis in EwS in three independent datasets. Our data may suggest that the role of *CDKN2A* may change across the clinical context of EwS, however, further study is necessary to validate the function of *CDKN2A* expression in EwS.

## Graphical abstract

We revisit *CDKN2A* to investigate its role as a potential prognostic biomarker in Ewing sarcoma using data from pre‐clinical models as well as clinical samples.

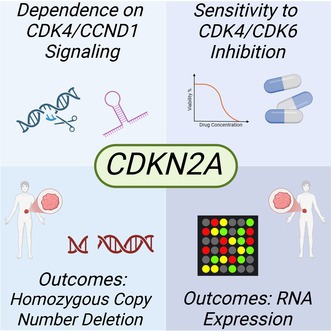

**Abbreviations**
95% CI: 95% confidence interval
ANOVA: analysis of variance
DepMap: Dependency Map
EwS: Ewing sarcoma
GDSC: Genomics of Drug Sensitivity in Cancer
GEO: Gene Expression Omnibus
HR: hazard ratio
IQR: interquartile range
IRB: Institutional Review Board
LYM: lymphoma
NB: neuroblastoma
OSt: osteosarcoma
RMS: rhabdomyosarcoma
SD: standard deviation

## 1 Introduction

Ewing sarcoma (EwS) is a rare and aggressive malignancy, which frequently affects children [1]. One of the few recurrent genomic variants in EwS is genomic copy number deletion of *CDKN2A* [2]. The *CDK4/CCND1‐RB1* pathway, regulated by *CDKN2A*, has emerged as a key molecular dependency in EwS [3, 4, 5]. Initial clinical reports suggested an association between *CDKN2A* homozygous copy number deletion and poor survival outcomes [2, 6, 7, 8, 9, 10, 11]; however, landmark studies published in 2014 found no significant survival difference based on genomic *CDKN2A* status [12, 13]. We recently reported that secondary genomic variants are likely not random in fusion driven sarcomas, including EwS, and may hold key biological meaning [14, 15, 16]. In this *in silico* study, we revisit *CDKN2A* as a prognostic biomarker in EwS using data from *in vitro* EwS models as well as clinical samples from patients with EwS. We present evidence of the biologic importance of *CDKN2A* downstream signaling in EwS and present evidence that suggests that *CDKN2A* may have a prognostic role in EwS.

## 2 Materials and methods

### 2.1 Dependency Map and project Achilles

Data from the Dependency Map (DepMap) and Project Achilles project, a CRISPR‐Cas9 and RNAi knockout database, were downloaded directly from the depmap portal (https://depmap.org/portal/download/custom/) [17]. Gene dependency effects for inactivation of genes downstream of *CDKN2A* including *CDK4*, *CDK6*, *CCND1*, *RB1*, *MDM2*, and *TP53* were included for analysis. The gene effect scores evaluate the effect size of knocking out/down a gene normalized against pan‐essential and nonessential genes. Negative scores represent genes essential for proliferation or survival.

### 2.2 Genomics of Drug Sensitivity in Cancer

Data, including DNA variants, mRNA expression, and palbociclib sensitivity were collected from the Genomics of Drug Sensitivity in Cancer (GDSC) database as previously described (http://cancerrxgene.org, downloaded August 22nd, 2019) [15, 18, 19].

### 2.3 DNA profiling of EwS tumors

Genomic profiling data of clinical Ewing sarcoma specimens were collected from publicly available datasets from Dermawan et al. [20], Nguyen et al. [21], and Gounder et al. [22]. Data from Dermawan et al. and Nguyen et al. were collected using cBioPortal [23, 24, 25]. Data from Gounder et al were downloaded directly from the source data published alongside their original report [22]. The aggregate frequency of *CDKN2A* genomic variants were collected from the available literature [2, 6, 7, 8, 9, 10, 12, 13, 26, 27, 28, 29, 30].

### 2.4 RNA profiling of EwS tumors

Data from independent datasets of EwS samples including GSE17618 (*n* = 24) [31], GSE63155 (*n* = 46) [32], and GSE63156 (*n* = 39) [32], were obtained from the Gene Expression Omnibus (GEO, https://www.ncbi.nlm.nih.gov/geo, downloaded March 3rd, 2024). GSE17618 used the Affymetrix Human Genome U133 Plus 2.0 microarray. GSE63155 and GSE63156 used the Affymetrix Human Exon 1.0 ST microarray. Only primary EwS samples were included. Overlapping probe IDs were aggregated by maximum probe expression.

### 2.5 Data analysis

This study was approved by the University of Florida Institutional Review Board (#IRB202101136). Data was analyzed in r v.4.1.1 or graphpad prism v.9.2.0. Descriptive statistics, Pearson correlation coefficient, Kruskal–Wallis tests, one‐sample *t*‐tests, Welch's *t*‐tests, analysis of variance (ANOVA), uncorrected Dunn's tests, and Mann–Whitney tests were used as appropriate. Survival analysis was tested using both log‐rank and Cox proportional hazard methods. Survival graphs were created using the Kaplan–Meier estimator. Unless otherwise stated, *P* values ≤ 0.05 were considered statistically significant.

## 3 Results

### 3.1 EwS are dependent on CDK4/CCND1 signaling

**Fig. 1:**
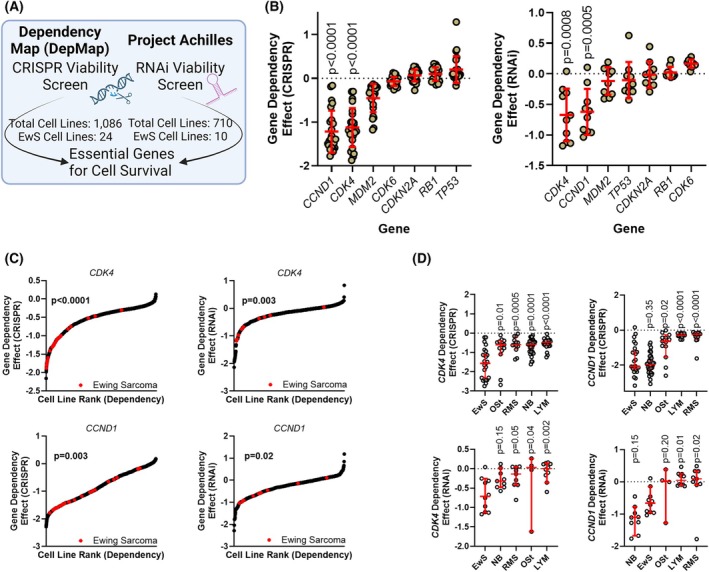
Dependency and sensitivity of *CDKN2A* pathway in EwS. (A) Data from the Dependency Map (DepMap) and Project Achilles project, a CRISPR‐Cas9 and RNAi knockout database. Gene effect scores evaluate the effect size of knocking out/down a gene normalized against pan‐essential and nonessential genes. Negative scores represent genes essential for proliferation. (B) Dependency of genes downstream of *CDKN2A* in EwS in DepMap (CRISPR, *n* = 24) and Project Achilles (RNAi, *n* = 10) datasets. Only *P* values < 0.05 by one‐sample *t*‐test with a discrepancy greater than 0.5 were included. Error bars indicate SD. (C) Ranking of dependency scores for *CDK4* and *CCND1* across cancer cell lines (DepMap, *n* = 1086; Project Achilles, *n* = 710). *P* values calculated by Kolmogorov–Smirnov test. (D) In comparison to other similar cancers, EwS cell lines demonstrated significantly greater dependence on *CCND1* and *CDK4* (DepMap: EwS *n* = 24, OSt *n* = 13, RMS *n* = 13, NB *n* = 36, LYM *n* = 23; Project Achilles: EwS *n* = 10, OSt *n* = 4, RMS *n* = 9, NB *n* = 9, LYM *n* = 10). *P* values generated using the Kruskal–Wallis and uncorrected Dunn's test and are comparison with the dependency effect of EwS. Error bars indicate 95% CI. LYM, lymphoma; NB, neuroblastoma; OSt, osteosarcoma; RMS, rhabdomyosarcoma.

To validate the dependence of EwS on the *CDKN2A* pathway, we collected data for 1086 cell lines (24 EwS lines) from DepMap and 710 cell lines (10 EwS lines) from Project Achilles directly from the DepMap portal [17]. For each dataset, we selected gene dependency effects for inactivation of genes downstream of *CDKN2A* (Fig. 1A). Within EwS cell lines, *CCND1* and *CDK4* were the only genes selected which resulted in consistent reduction in viability when inactivated in both the DepMap and Project Achilles datasets (Fig. 1B). This data suggests that a bottleneck for EwS proliferation or survival is dependent on active signaling at the CDK4/Cyclin D1 complex, immediately downstream of and regulated by *CDKN2A*. In comparison with other cancer cell lines, EwS lines demonstrated increased dependency for *CCND1* and *CDK4* in both datasets (Fig. 1C). Specifically, in comparison to the most common pediatric sarcomas and small round blue cell tumors, EwS lines demonstrated significant dependence on *CCND1* and *CDK4* (Fig. 1D) [33]. Dependance on *CDK4* and *CCND1* was not associated with the genomic *CDKN2A* status in EwS cell lines (Fig. S1).

### 3.2 EwS cell lines are highly sensitive to CDK4/CDK6 inhibition

**Fig. 2:**
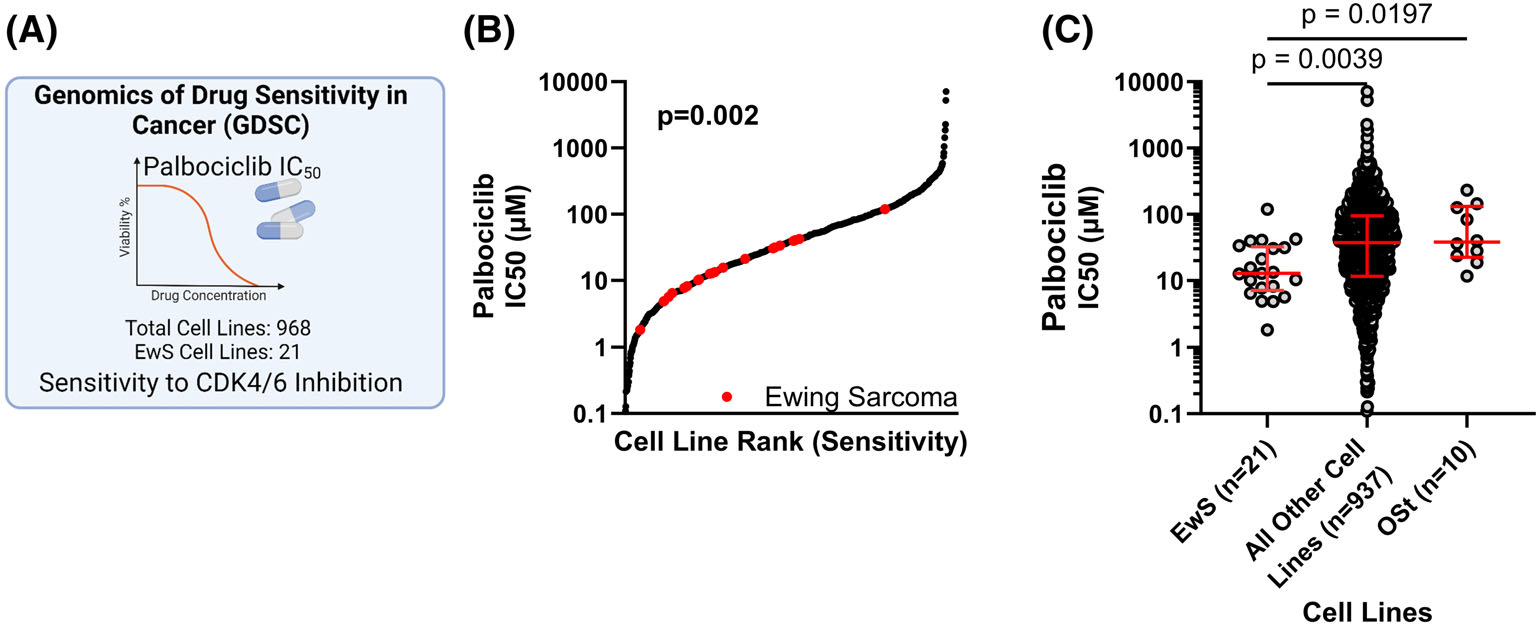
Dependency and sensitivity of *CDKN2A* pathway in EwS. (A) Association between genomic markers and drug sensitivity were collected directly from the Genomics of Drug Sensitivity in Cancer (GDSC) database (*n* = 968). (B) Ranking of cell line IC50s for palbociclib. *P* values calculated by Kolmogorov–Smirnov test. (C) In comparison to osteosarcoma (*n* = 10) or all other cell lines (*n* = 937), EwS cell lines (*n* = 21) demonstrated greater sensitivity to palbociclib. *P* value generated using the Mann–Whitney test. Error bars indicate IQR. OSt, osteosarcoma.

The potential importance of *CCND1* and *CDK4* activity can further be demonstrated through the *in vitro* sensitivity of cell lines to CDK4/6 inhibitors. Using data from the Genomics of Drug Sensitivity in Cancer (GDSC) database, we demonstrate that EwS cell lines are sensitive to treatment with the CDK4/6 inhibitor palbociclib (Fig. 2A). Across 968 cell lines, representing 56 tissue types, EwS lines demonstrated increased sensitivity to palbociclib (Fig. 2B). Specifically, in comparison to osteosarcoma, EwS lines were significantly more sensitive to CDK4/6inhibition with palbociclib (Fig. 2C). Sensitivity to palbociclib in EwS cell lines was not dependent on genomic *CDKN2A* status (Fig. S2). Taken together, data from DepMap (3.1), Project Achilles (3.1), and GDSC clearly demonstrate a vital signaling bottleneck immediately downstream of *CDKN2A* at *CDK4*/*CCND1*.

### 3.3 The clinical significance of CDKN2A homozygous copy number deletion is unclear

**Fig. 3:**
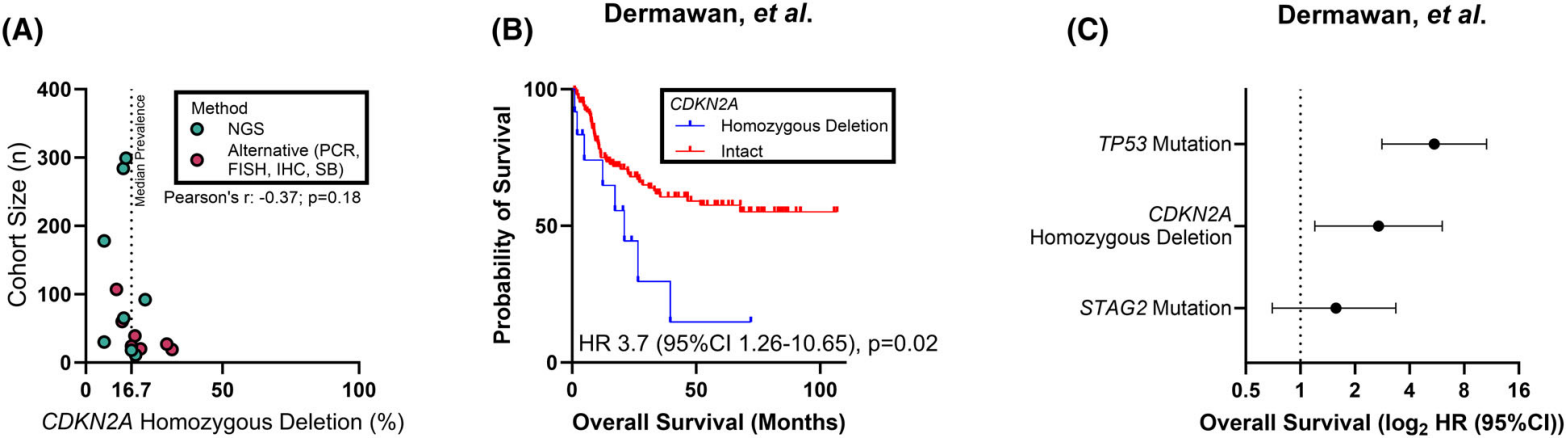
Genomic loss of *CDKN2A* is associated with poor overall survival in EwS. (A) Homozygous copy number deletion of CDKN2A has been reported in 6–32% of EwS, with a median prevalence in selected studies of 16.7%. (B, C) Clinico‐genomic data from Dermawan et al. Kaplan–Meier plot of overall survival for patients with EwS stratified by *CDKN2A* status (B). Multivariable model for overall survival with molecular covariates *TP53* and *STAG2* (C). Error bars indicate 95% CI. 95% CI, 95% confidence interval; HR, hazard ratio.

In patient samples, homozygous copy number deletion of *CDKN2A* is one of the most prevalent genomic variants identified in EwS. To estimate the prevalence of homozygous copy number deletion of *CDKN2A* in EwS we collected data from 15 clinical studies. Deletion of *CDKN2A* was identified in 6–32% of EwS, with a median prevalence of approximately 16.7% (Fig. 3A) [2, 6, 7, 8, 9, 10, 12, 13, 20, 22, 26, 27, 28, 29, 30]. To further assess the correlation between *CDKN2A* homozygous copy number deletion and overall survival in EwS, we collected publicly available genomic sequencing data generated from 178 EwS patients [20]. *CDKN2A* homozygous copy number deletion was identified in 12 tumors (6.7%) and was associated with reduced overall survival (hazard ratio [HR] 3.7, 95% confidence interval 1.26–10.65, *P* = 0.02, Fig. 3B). In a multivariate analysis with *STAG2* and *TP53* mutations, which have also been associated with poor overall survival in EwS [12], *CDKN2A* homozygous copy number deletion remains significantly associated with overall survival (Fig. 3C).

### 3.4 Elevated expression of CDKN2A is associated with poor overall survival

**Fig. 4:**
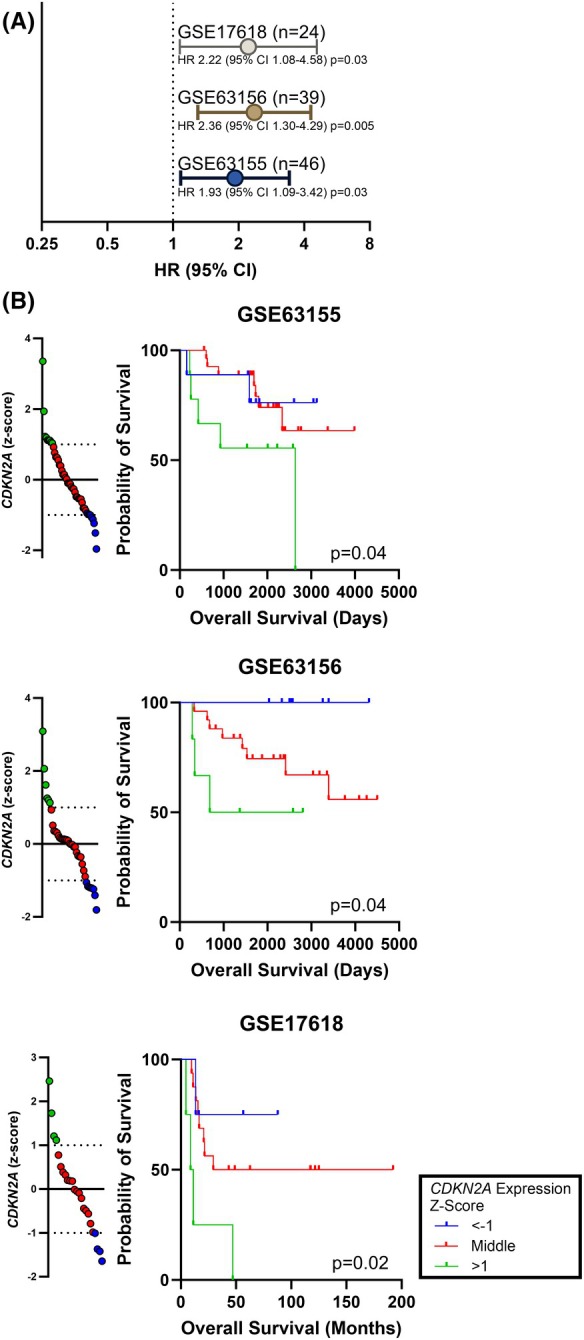
Elevated *CDKN2A* expression is associated with poor overall survival in EwS. Data from independent datasets of primary EwS samples including GSE17618 (*n* = 24), GSE63155 (*n* = 46), and GSE63156 (*n* = 39), were obtained from the Gene Expression Omnibus (GEO). (A) Expression of *CDKN2A* is associated with poor overall survival by Cox proportional hazard regression with *CDKN2A* as a continuous variable (HR is increased risk for each *z*‐score increase in *CDKN2A* expression) for three independent clinical datasets. Error bars indicate 95% CI. (B) For each clinical dataset, Kaplan–Meier curves were generated by categorizing samples by *z*‐score, with a *z*‐score > 1 as *CDKN2A* high expression and a *z*‐score < −1 as *CDKN2A* low expression. 95% CI, 95% confidence interval; HR, hazard ratio.

While significant efforts have been spent to understand the role of homozygous copy number deletion of *CDKN2A* in EwS, little published data testing the role of intact *CDKN2A* expression, which is present in 68–94% of EwS, exists. We therefore investigated the clinical association between *CDKN2A* RNA expression and overall survival using three publicly available datasets for a total of 109 patients with EwS [31, 32]. In each dataset, we identified an association between high expression of *CDKN2A* and reduced overall survival. As a continuous predictor of overall survival, increases of *CDKN2A* by a *z*‐score of 1 represented an increased HR between 1.9 and 2.4 across all three datasets (all *P* < 0.05, Fig. 4A). We then categorized samples by *z*‐score, with a *z*‐score > 1 as *CDKN2A* high expression and a *z*‐score < −1 as *CDKN2A* low expression. For each dataset, a *CDKN2A* high expression was associated with poor overall survival (Fig. 4B).

## 4 Discussion

In this study, we present the potential oncogenic essentiality of *CDKN2A* dysregulation and sustained downstream *CDK4*/*CCND1* activity and complex clinical associations of *CDKN2A* dysregulation in EwS, confirming prior reports. Furthermore, we demonstrate that treatment with synthetic *CDKN2A*, in the form of CDK4/6 inhibitors, significantly reduces cell viability in EwS compared to other cancers. It is important to consider the difference between a molecular dependency which is and is not clinically targetable. Despite a dependence on CDK4 and Cyclin D1 activity and the efficacy of CDK4/6 inhibitors in pre‐clinical models of EwS, [3, 4, 34, 35, 36] clinical benefit from these agents has not been reported in patients with EwS [37]. Further studies of CDK4/6 inhibitors in EwS are currently being conducted (Table S1). There is still a need to further dissect the differential response to this therapeutic strategy between pre‐clinical models and actual patient tumors in the relapsed or refractory setting where they are being studied. In a recent pre‐print, Funk et al. [36] reported that chromosome 8 gain led to increased expression of translation initiation factor 4E‐BP1, increased proliferative signaling, and ultimately sensitivity to CDK4/6 inhibitors in EwS. Additionally, sensitivity to CDK2 inhibition has been demonstrated by Musa et al. [38] to limit the proliferation of EwS. EwS tumors are highly proliferative, with the driving *EWSR1*::*FLI1*/*ERG* translocation directly altering multiple proliferative pathways. Further study to dissect individual drivers of proliferation in EwS is necessary.

We also assessed the clinical consequences of *CDKN2A* in EwS. The evidence for the prognostic value of *CDKN2A* homozygous copy number deletion in the literature is varied with multiple retrospective studies [2, 6, 7, 8, 9, 10] and one meta‐analysis [11] suggesting a negative prognostic effect (tumor samples coming from multiple treatment time points) and two prospective studies suggesting no significant clinical association (primarily taken from tumors at diagnosis) [12, 13]. In a large retrospective dataset, we found evidence that *CDKN2A* homozygous copy number deletion was a negative prognostic marker. One possible driver of these inconsistent results is the clinical heterogeneity of patients included in retrospective studies. A second possibility is that patients receiving clinical genomic sequencing of their tumor, as is the case for many of the datasets cited here, are more likely to include refractory, recurrent, or metastatic tumors compared to tumors which are never sent for genomic sequencing. Therefore, with the data available, it is not possible to know when *CDKN2A* homozygous copy number deletion occurs in the oncogenic course of EwS, or if the effect on overall survival is due to therapy response, metastatic potential, or other mechanisms of aggressive disease.

Inversely, we found that high expression of *CDKN2A* was a negative prognostic biomarker at diagnosis in EwS in three independent datasets. This finding has previously been reported in an independent set of 33 patients with EwS which associated elevated expression *CDKN2A* with a reduced event‐free survival [39]. This finding is somewhat limited due to the use of RNA microarrays to generate RNA expression in these cohorts (Fig. S3). These tools are limited in their dynamic range, resulting in an inability to identify complete lack of expression or to differentiate between low and moderately expressed genes. Further research using methods with greater dynamic range, such as RT‐PCR or RNA‐seq, is necessary. Our initial hypothesis for the mechanism of high expression of *CDKN2A* as a negative prognostic biomarker at diagnosis in EwS was that elevated *CDKN2A* expression may lead to decreased tumor cell growth rate, decreased sensitivity to chemotherapeutics, and therefore poor clinical outcomes. An exploratory analysis of the GDSC data demonstrates that in EwS cell lines *CDKN2A* expression may decrease growth rate and chemotherapy sensitivity (Fig. S4).

Taken together, this data may suggest that in the early stage of EwS, and during frontline therapy, elevated expression of *CDKN2A* may be beneficial to EwS survival. Additionally, in the early (i.e. prior to diagnosis) and later (i.e. prior to relapse) stages of clinical care, *CDKN2A* homozygous copy number deletion may be beneficial to EwS tumors due to the proliferative advantage conferred. Further study is necessary to validate the function of *CDKN2A* expression in this context. The data here should be considered as hypothesis generating until further studies can validate the findings functionally and in clinical samples.

There are several limitations to this study, which should be considered when interpreting our results. First, this study is limited by the inherent nature of retrospective studies. Small datasets of this rare sarcoma further reduce the potential external validity of the findings of this study. Like many cancers, particularly true of rare cancers, pre‐clinical EwS models are not perfect surrogates of EwS in the clinic and this limitation has stymied drug development in an era of immunotherapy advances [10, 26, 29, 40]. Finally, it is clear that *CDKN2A* alone does not explain the total biological or clinical heterogeneity present in EwS. Further research is necessary to understand the role of the *CDKN2A* pathway in the greater molecular context of EwS.

Taken together, the data presented here suggests that *CDKN2A* may play a key molecular role in EwS. While it remains unclear what the consequences of *CDKN2A* suppression are clinically, the role is likely dependent on the clinical context of the disease. Future studies of *CDKN2A* in EwS should focus on clinical contexts, specific to diagnosis, initial treatment response, and development of relapse and metastasis as well as the multi‐omic dysregulation of *CDKN2A* identified in EwS, in order to provide more clarity to the role of this complex biomarker.

## 5 Conclusions

Our findings suggest that *CDKN2A* dysregulation is a key molecular characteristic in EwS. Furthermore, the prognostic value of *CDKN2A* in EwS may be context dependent. Future studies should consider *CDKN2A* as a biomarker in EwS in relation to the clinical treatment course of the patient.

## Conflict of interest

The authors declare no Conflict of interest.

## Author contributions

NDS designed the research. AP, VS, JKL, EJS, and NDS analyzed the data. AP, VS, EN, JAL, LAE, PC‐C, JKL, EJS, and NDS wrote the paper.

### Peer review

The peer review history for this article is available at https://www.webofscience.com/api/gateway/wos/peer‐review/10.1002/1878‐0261.70008.

## Code availability

Data was analyzed in r v.4.1.1 or graphpad prism v.9.2.0. No custom code was used for this analysis.

## Supporting information


**Fig. S1.** Dependency for *CDK4* and *CCND1*.
**Fig. S2.** Palbociclib IC50.
**Fig. S3.** Distribution in expression of *CDKN2A*.
**Fig. S4.** Exploratory analysis of the GDSC.
**Table S1.** Clinical trials with CDK4/6 inhibitors accepting patients with Ewing sarcoma.

## Data Availability

Data from the DepMap and Project Achilles project are available from the depmap portal (https://depmap.org/portal/download/custom/) [17]. The gene expression data used in this study are available from the Gene Expression Omnibus repository under accession numbers GSE17618 [31], GSE63155 [32], and GSE63156 [32]. The drug sensitivity data are accessible from the Genomics of Drug Sensitivity in Cancer Database (https://www.cancerrxgene.org/) [15, 18, 19]. Genomic profiling data of clinical Ewing sarcoma specimens were collected from publicly available datasets from Dermawan et al. [20], Nguyen et al. [21], and Gounder et al. [22]. Data from Dermawan et al. and Nguyen et al. were collected using cBioPortal (https://www.cbioportal.org/) [23, 24, 25]. Data from Gounder et al. were downloaded directly from the source data published alongside their original report (https://www.nature.com/articles/s41467‐022‐30496‐0#data‐availability) [22].

## References

[1] Grünewald TGP , Cidre‐Aranaz F , Surdez D , Tomazou EM , de Álava E , Kovar H , et al. Ewing sarcoma. Nat Rev Dis Primers. 2018;4:5. 29977059 10.1038/s41572-018-0003-x

[2] Huang HY , Illei PB , Zhao Z , Mazumdar M , Huvos AG , Healey JH , et al. Ewing sarcomas with p53 mutation or p16/p14ARF homozygous deletion: a highly lethal subset associated with poor chemoresponse. J Clin Oncol. 2005;23:548–558. 15659501 10.1200/JCO.2005.02.081

[3] Kennedy AL , Vallurupalli M , Chen L , Crompton B , Cowley G , Vazquez F , et al. Functional, chemical genomic, and super‐enhancer screening identify sensitivity to cyclin D1/CDK4 pathway inhibition in Ewing sarcoma. Oncotarget. 2015;6:30178–30193. 26337082 10.18632/oncotarget.4903 PMC4745789

[4] Guenther LM , Dharia NV , Ross L , Conway A , Robichaud AL , Catlett JL II , et al. A combination CDK4/6 and IGF1R inhibitor strategy for Ewing sarcoma. Clin Cancer Res. 2019;25:1343–1357. 30397176 10.1158/1078-0432.CCR-18-0372 PMC6498855

[5] Miller HE , Gorthi A , Bassani N , Lawrence LA , Iskra BS , Bishop AJR . Reconstruction of Ewing sarcoma developmental context from mass‐scale transcriptomics reveals characteristics of EWSR1‐FLI1 permissibility. Cancer. 2020;12(4):948. 10.3390/cancers12040948 PMC7226175 32290418

[6] López‐Guerrero JA , Pellín A , Noguera R , Carda C , Llombart‐Bosch A . Molecular analysis of the 9p21 locus and p53 genes in Ewing family tumors. Lab Invest. 2001;81:803–814. 11406642 10.1038/labinvest.3780290

[7] Maitra A , Roberts H , Weinberg AG , Geradts J . Aberrant expression of tumor suppressor proteins in the Ewing family of tumors. Arch Pathol Lab Med. 2001;125:1207–1212. 11520274 10.5858/2001-125-1207-AEOTSP

[8] Wei G , Antonescu CR , de Alava E , Leung D , Huvos AG , Meyers PA , et al. Prognostic impact of ink4a deletion in Ewing sarcoma. Cancer. 2000;89:793–799. 10951342 10.1002/1097-0142(20000815)89:4<793::aid-cncr11>3.0.co;2-m

[9] Tsuchiya T , Sekine K , Hinohara S , Namiki T , Nobori T , Kaneko Y . Analysis of the p16ink4, p14arf, p15, tp53, and MDM2 genes and their prognostic implications in osteosarcoma and Ewing sarcoma. Cancer Genet Cytogenet. 2000;120:91–98. 10942797 10.1016/s0165-4608(99)00255-1

[10] Kovar H , Jug G , Aryee DN , Zoubek A , Ambros P , Gruber B , et al. Among genes involved in the rb dependent cell cycle regulatory cascade, the p16 tumor suppressor gene is frequently lost in the Ewing family of tumors. Oncogene. 1997;15:2225–2232. 9393981 10.1038/sj.onc.1201397

[11] Honoki K , Stojanovski E , McEvoy M , Fujii H , Tsujiuchi T , Kido A , et al. Prognostic significance of p16 INK4a alteration for Ewing sarcoma: a meta‐analysis. Cancer. 2007;110:1351–1360. 17661343 10.1002/cncr.22908

[12] Tirode F , Surdez D , Ma X , Parker M , le Deley MC , Bahrami A , et al. Genomic landscape of Ewing sarcoma defines an aggressive subtype with co‐association of STAG2 and TP53 mutations. Cancer Discov. 2014;4:1342–1353. 25223734 10.1158/2159-8290.CD-14-0622 PMC4264969

[13] Lerman DM , Monument MJ , McIlvaine E , Liu XQ , Huang D , Monovich L , et al. Tumoral TP53 and/or CDKN2A alterations are not reliable prognostic biomarkers in patients with localized Ewing sarcoma: a report from the children's oncology group. Pediatr Blood Cancer. 2015;62:759–765. 25464386 10.1002/pbc.25340 PMC4376595

[14] Walker V , Jin DX , Millis SZ , Nasri E , Corao‐Uribe DA , Tan AC , et al. Gene partners of the EWSR1 fusion may represent molecularly distinct entities. Transl Oncol. 2023;38:101795. 37797367 10.1016/j.tranon.2023.101795 PMC10593575

[15] Seligson ND , Maradiaga RD , Stets CM , Katzenstein HM , Millis SZ , Rogers A , et al. Multiscale‐omic assessment of EWSR1‐NFATc2 fusion positive sarcomas identifies the mtor pathway as a potential therapeutic target. NPJ Precis Oncol. 2021;5:43. 34021224 10.1038/s41698-021-00177-0 PMC8140100

[16] Seligson ND , Awasthi A , Millis SZ , Turpin BK , Meyer CF , Grand'Maison A , et al. Common secondary genomic variants associated with advanced epithelioid hemangioendothelioma. JAMA Netw Open. 2019;2:e1912416. 31577358 10.1001/jamanetworkopen.2019.12416 PMC6777396

[17] Pacini C , Duncan E , Gonçalves E , Gilbert J , Bhosle S , Horswell S , et al. A comprehensive clinically informed map of dependencies in cancer cells and framework for target prioritization. Cancer Cell. 2024;42:301–316.e9. 38215750 10.1016/j.ccell.2023.12.016

[18] Yang W , Soares J , Greninger P , Edelman EJ , Lightfoot H , Forbes S , et al. Genomics of drug sensitivity in cancer (GDSC): a resource for therapeutic biomarker discovery in cancer cells. Nucleic Acids Res. 2013;41:D955–D961. 23180760 10.1093/nar/gks1111 PMC3531057

[19] Ghandi M , Huang FW , Jané‐Valbuena J , Kryukov GV , Lo CC , McDonald ER III , et al. Next‐generation characterization of the cancer cell line encyclopedia. Nature. 2019;569:503–508. 31068700 10.1038/s41586-019-1186-3 PMC6697103

[20] Dermawan JK , Slotkin E , Tap WD , Meyers P , Wexler L , Healey J , et al. Chromoplexy is a frequent early clonal event in ewsr1‐rearranged round cell sarcomas that can be detected using clinically validated targeted sequencing panels. Cancer Res. 2024;84:1504–1516. 38335254 10.1158/0008-5472.CAN-23-2573 PMC11648190

[21] Nguyen B , Fong C , Luthra A , Smith SA , RG DN , Nandakumar S , et al. Genomic characterization of metastatic patterns from prospective clinical sequencing of 25,000 patients. Cell. 2022;185:563–575.e11. 35120664 10.1016/j.cell.2022.01.003 PMC9147702

[22] Gounder MM , Agaram NP , Trabucco SE , Robinson V , Ferraro RA , Millis SZ , et al. Clinical genomic profiling in the management of patients with soft tissue and bone sarcoma. Nat Commun. 2022;13:3406. 35705558 10.1038/s41467-022-30496-0 PMC9200814

[23] Cerami E , Gao J , Dogrusoz U , Gross BE , Sumer SO , Aksoy BA , et al. The cBio cancer genomics portal: an open platform for exploring multidimensional cancer genomics data. Cancer Discov. 2012;2:401–404. 22588877 10.1158/2159-8290.CD-12-0095 PMC3956037

[24] Gao J , Aksoy BA , Dogrusoz U , Dresdner G , Gross B , Sumer SO , et al. Integrative analysis of complex cancer genomics and clinical profiles using the cbioportal. Sci Signal. 2013;6:pl1. 23550210 10.1126/scisignal.2004088 PMC4160307

[25] de Bruijn I , Kundra R , Mastrogiacomo B , Tran TN , Sikina L , Mazor T , et al. Analysis and visualization of longitudinal genomic and clinical data from the AACR project genie biopharma collaborative in cBioPortal. Cancer Res. 2023;83:3861–3867. 37668528 10.1158/0008-5472.CAN-23-0816 PMC10690089

[26] Crompton BD , Stewart C , Taylor‐Weiner A , Alexe G , Kurek KC , Calicchio ML , et al. The genomic landscape of pediatric Ewing sarcoma. Cancer Discov. 2014;4:1326–1341. 25186949 10.1158/2159-8290.CD-13-1037

[27] Worst BC , van Tilburg CM , Balasubramanian GP , Fiesel P , Witt R , Freitag A , et al. Next‐generation personalised medicine for high‐risk paediatric cancer patients – the inform pilot study. Eur J Cancer. 2016;65:91–101. 27479119 10.1016/j.ejca.2016.06.009

[28] Wong M , Mayoh C , Lau LMS , Khuong‐Quang DA , Pinese M , Kumar A , et al. Whole genome, transcriptome and methylome profiling enhances actionable target discovery in high‐risk pediatric cancer. Nat Med. 2020;26:1742–1753. 33020650 10.1038/s41591-020-1072-4

[29] Brohl AS , Solomon DA , Chang W , Wang J , Song Y , Sindiri S , et al. The genomic landscape of the Ewing sarcoma family of tumors reveals recurrent STAG2 mutation. PLoS Genet. 2014;10:e1004475. 25010205 10.1371/journal.pgen.1004475 PMC4091782

[30] Wu L , Yao H , Chen H , Wang A , Guo K , Gou W , et al. Landscape of somatic alterations in large‐scale solid tumors from an Asian population. Nat Commun. 2022;13:4264. 35871175 10.1038/s41467-022-31780-9 PMC9308789

[31] Savola S , Klami A , Myllykangas S , Manara C , Scotlandi K , Picci P , et al. High expression of complement component 5 (c5) at tumor site associates with superior survival in Ewing's sarcoma family of tumour patients. ISRN Oncol. 2011;2011:168712. 22084725 10.5402/2011/168712 PMC3196920

[32] Volchenboum SL , Andrade J , Huang L , Barkauskas DA , Krailo M , Womer RB , et al. Gene expression profiling of Ewing sarcoma tumors reveals the prognostic importance of tumor‐stromal interactions: a report from the children's oncology group. J Pathol Clin Res. 2015;1:83–94. 26052443 10.1002/cjp2.9 PMC4457396

[33] Gregorio A , Corrias MV , Castriconi R , Dondero A , Mosconi M , Gambini C , et al. Small round blue cell tumours: diagnostic and prognostic usefulness of the expression of b7‐h3 surface molecule. Histopathology. 2008;53:73–80. 18613926 10.1111/j.1365-2559.2008.03070.x PMC2658025

[34] Dowless M , Lowery CD , Shackleford T , Renschler M , Stephens J , Flack R , et al. Abemaciclib is active in preclinical models of Ewing sarcoma via multipronged regulation of cell cycle, dna methylation, and interferon pathway signaling. Clin Cancer Res. 2018;24:6028–6039. 30131386 10.1158/1078-0432.CCR-18-1256 PMC6279561

[35] Murakami T , Singh AS , Kiyuna T , Dry SM , Li Y , James AW , et al. Effective molecular targeting of CDK4/6 and IGF‐1R in a rare FUS‐ERG fusion CDKN2a‐deletion doxorubicin‐resistant Ewing's sarcoma patient‐derived orthotopic xenograft (PDOX) nude‐mouse model. Oncotarget. 2016;7:47556–47564. 27286459 10.18632/oncotarget.9879 PMC5216960

[36] Funk CM , Ehlers AC , Orth MF , Aljakouch K , Li J , Hölting TLB , et al. Chromosome 8 gain drives cancer progression by hijacking the translation factor 4E‐BP1 sensitizing for targeted CDK4/6 inhibition. bioRxiv. 2022.

[37] Shulman DS , Merriam P , Choy E , Guenther LM , Cavanaugh KL , Kao PC , et al. Phase 2 trial of palbociclib and ganitumab in patients with relapsed Ewing sarcoma. Cancer Med. 2023;12:15207–15216. 37306107 10.1002/cam4.6208 PMC10417097

[38] Musa J , Cidre‐Aranaz F , Aynaud MM , Orth MF , Knott MML , Mirabeau O , et al. Cooperation of cancer drivers with regulatory germline variants shapes clinical outcomes. Nat Commun. 2019;10:4128. 31511524 10.1038/s41467-019-12071-2 PMC6739408

[39] Brownhill SC , Taylor C , Burchill SA . Chromosome 9p21 gene copy number and prognostic significance of p16 in esft. Br J Cancer. 2007;96:1914–1923. 17533400 10.1038/sj.bjc.6603819 PMC2359978

[40] Morales E , Olson M , Iglesias F , Dahiya S , Luetkens T , Atanackovic D . Role of immunotherapy in Ewing sarcoma. J Immunother Cancer. 2020;8(2):e000653. 33293354 10.1136/jitc-2020-000653 PMC7725096

