# Supplementary material for: Revisiting CDKN2A dysregulation in Ewing sarcoma
Source: Mol Oncol. 2025 Mar 13;19(4):994–1001. doi: 10.1002/1878-0261.70008 (PMC11977643; doi:10.1002/1878-0261.70008)
Supplement: Supplementary file 1 — Fig. S1. Dependency for CDK4 and CCND1. Fig. S2. Palbociclib IC50. Fig. S3. Distribution in expression of CDKN2A. Fig. S4. Exploratory analysis of the GDSC. Table S1. Clinical trials with CDK4/6 inhibitors accepting patients with Ewing sarcoma. [file MOL2-19-994-s001.docx]

Supplementary Material

- Supplemental Figure 1: Dependency for *CDK4* and *CCND1*
- Supplemental Figure 2: Palbociclib IC50
- Supplemental Figure 3: Distribution in expression of *CDKN2A*
- Supplemental Figure 4: Exploratory Analysis of the GDSC
- Supplemental Table 1: Clinical Trials With CDK4/6 Inhibitors Accepting Patients with Ewing Sarcoma

**Supplemental Figure 1: Dependency for *CDK4* and *CCND1***

**
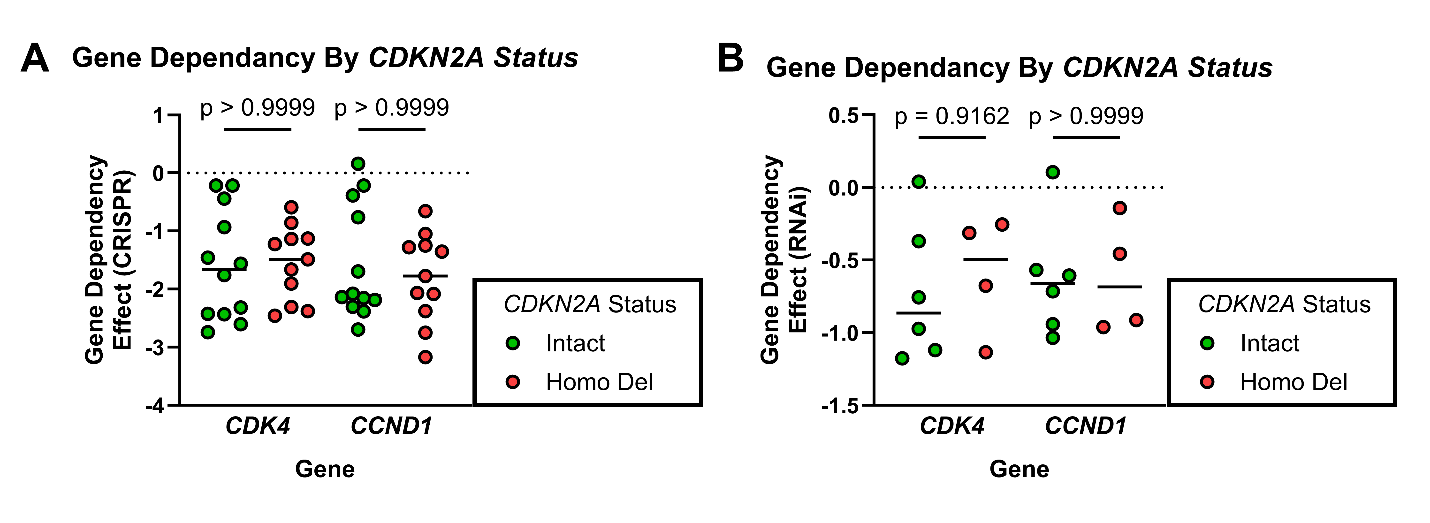
**

Dependency for *CDK4* and *CCND1* in EwS in DepMap (CRISPR, **A**) and Project Achilles (RNAi, **B**) datasets stratified by *CDKN2A* status. P values generated using the Kruskal-Wallis and uncorrected Dunn's test.

**Supplemental Figure 2: Palbociclib IC50**

**
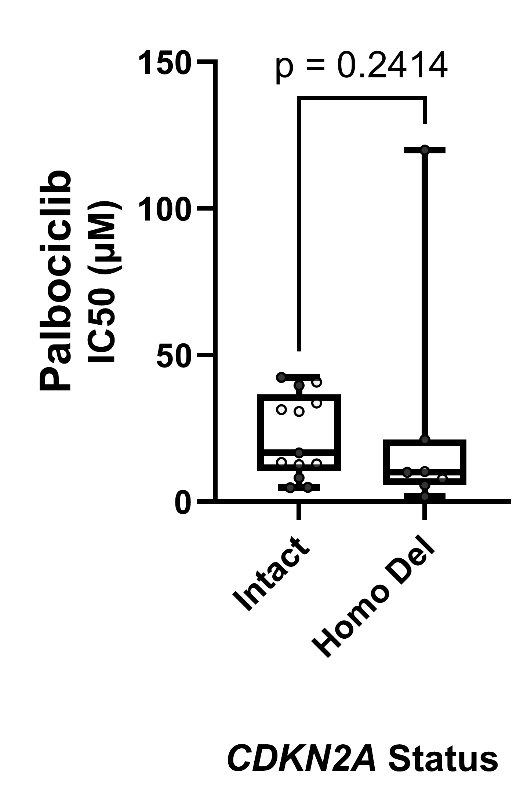
**

EwS cell line IC50s for palbociclib stratified by *CDKN2A* status. P value generated using the Mann-Whitney test.

**Supplemental Figure 3: Distribution in expression of *CDKN2A***

**
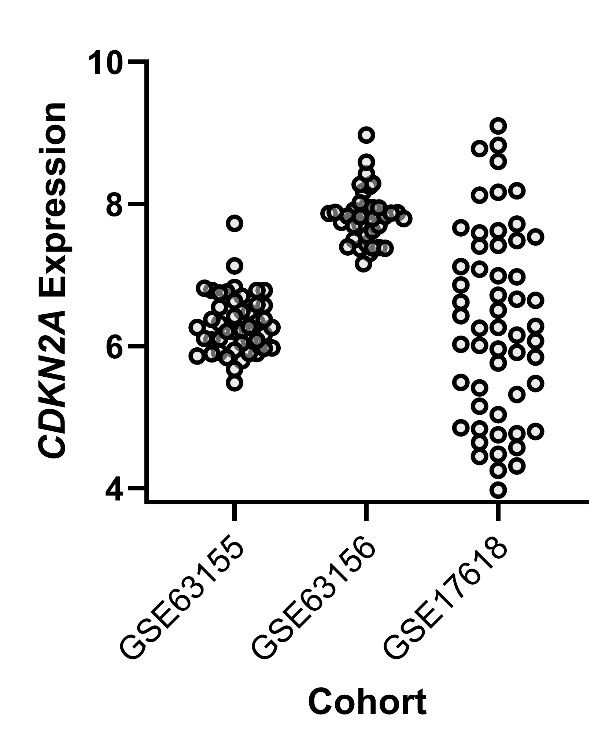
**

Distribution in expression of *CDKN2A* as measured by RNA microarray (GSE63155 and GSE63156: Affymetrix Human Exon 1.0 ST Array, GSE17618: Affymetrix Human Genome U133 Plus 2.0 Array) across three clinical cohorts of patients with EwS.

**Supplemental Figure 4: Exploratory Analysis of the GDSC**


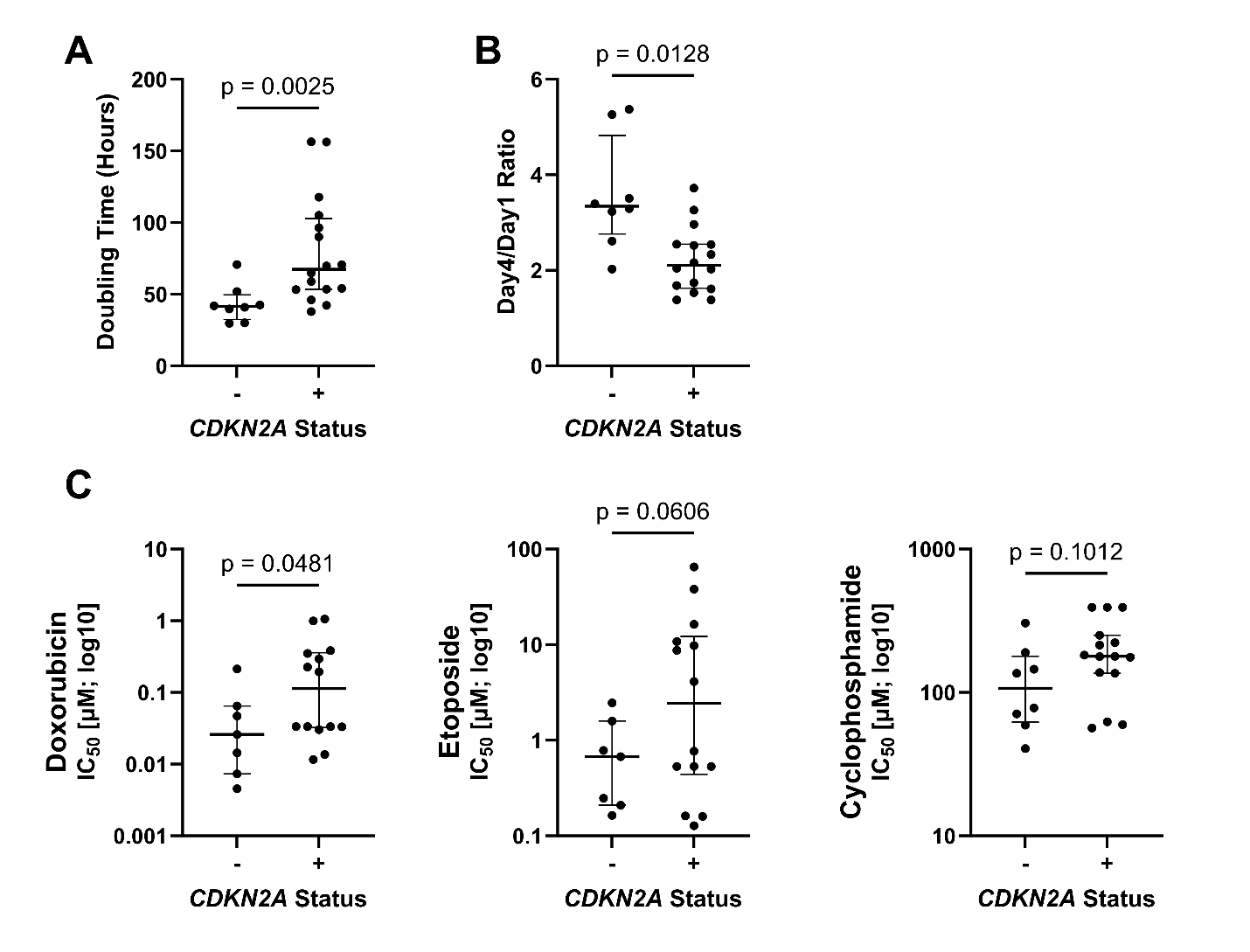


**A**) Cell doubling time for EwS cell lines by *CDKN2A* status. **B**) Cell number ration from days 4 and 1 for EwS cell lines by *CDKN2A* status. **C**) IC50s for EwS cell lines by *CDKN2A* status. All p values by Welch’s t test. Lines indicate median and interquartile range.

**Supplemental Table 1: Clinical Trials With CDK4/6 Inhibitors Accepting Patients with Ewing Sarcoma**

| Supplemental Table 1: Clinical Trials With CDK4/6 Inhibitors Accepting Patients with Ewing Sarcoma | | | |
| --- | --- | --- | --- |
| Study Title | NCT Identifier | Recruitment Status | Results |
| Palbociclib + Ganitumab In Ewing Sarcoma | NCT04129151 | Completed | RR 0%^1^ |
| Abemaciclib in Children With DIPG or Recurrent/Refractory Solid Tumors (AflacST1501) | NCT02644460 | Completed | - |
| Study Of Palbociclib Combined With Chemotherapy In Pediatric Patients With Recurrent/Refractory Solid Tumors | NCT03709680 | Active, not recruiting | - |
| Palbociclib in Treating Patients With Relapsed or Refractory Rb Positive Advanced Solid Tumors, Non-Hodgkin Lymphoma, or Histiocytic Disorders With Activating Alterations in Cell Cycle Genes (A Pediatric MATCH Treatment Trial) | NCT03526250 | Active, not recruiting | - |
| Targeted Therapy Directed by Genetic Testing in Treating Pediatric Patients With Relapsed or Refractory Advanced Solid Tumors, Non-Hodgkin Lymphomas, or Histiocytic Disorders (The Pediatric MATCH Screening Trial) | NCT03155620 | Recruiting | - |
| A Study of Abemaciclib (LY2835219) in Combination With Other Anti-Cancer Treatments in Children and Young Adult Participants With Solid Tumors, Including Neuroblastoma | NCT04238819 | Recruiting | - |
| CAMPFIRE: A Study of Abemaciclib (LY2835219) in Participants With Ewing's Sarcoma | NCT05440786 | Recruiting | - |

RR: Response rate

1 Shulman, D. S. *et al.* Phase 2 trial of palbociclib and ganitumab in patients with relapsed Ewing sarcoma. *Cancer Med* **12**, 15207-15216 (2023). <https://doi.org/10.1002/cam4.6208>
